# Bowel Management in Hirschsprung Disease—Pre-, Peri- and Postoperative Care for Primary Pull-Through

**DOI:** 10.3390/children11050588

**Published:** 2024-05-13

**Authors:** Judith Lindert, Felix Schulze, Stefanie Märzheuser

**Affiliations:** Department of Paediatric Surgery, Paediatric Colorectal Center Rostock, University Hospital Rostock, Ernst-Heydemann Str. 8, 18057 Rostock, Germany; felix.schulze@med.uni-rostock.de (F.S.); stefanie.maerzheuser@med.uni-rostock.de (S.M.)

**Keywords:** bowel management, Hirschsprung disease, rectal washout, transanal irrigation, preparation for surgery, distal outlet obstruction, fecal incontinence

## Abstract

(1) Background: Bowel management contributes throughout the pathway of care for children with Hirschsprung. Preoperative bowel management prepares the child and family for the pull-through surgery. Perioperative bowel management supports early recovery and tailored bowel management in the follow-up supports the achievement of social continence. (2) Methods: We conducted a cross-sectional assessment of our institutional bowel management program to illustrate the pre-, peri- and postoperative bowel management strategies. (3) Results: A total of 31 children underwent primary pull-through, 23 without a stoma and 8 with a stoma, at a median age of 9 months. All children without a stoma were prepared for surgery by using rectal irrigations. Children with a stoma were prepared for surgery with a transfer of stoma effluent. Transanal irrigation supported early recovery. (4) Conclusions: Bowel management is a key pillar of the management of children with Hirschsprung disease. Incorporating bowel management in the pathway of care facilitates primary pull-through and supports perioperative recovery.

## 1. Introduction

Hirschsprung disease (HD) is a congenital malformation of the intestine in which the intestinal wall lacks ganglion cells. This results in a chronic intestinal motility disorder, equivalent to an intestinal obstruction. HD is a rare disease with an incidence of 1 in 2500 live births. A total of 90% of patients with HD are diagnosed in the neonatal/early infant period. The majority (approximately 80%) of cases involve the rectosigmoid [1].

The main treatment goal for HD is to achieve social continence. In addition to specialized surgery, patients require preparation for reconstructive surgery and a standardized but individualized long-term follow-up with bowel management. Thus, bowel management accompanies HD patients from the neonatal period through surgical reconstruction and right through to long-term follow-up. Living with a chronic disease such as HD can have a long-term impact on the psycho-social health of the affected children and their families [2,3]. Therefore, patient-centered, comprehensive care is important for the promotion of coping mechanisms.

To avoid obstructive symptoms and prevent unnecessary episodes of Hirschsprung-associated enterocolitis (HAEC), effective and adequate decompression must be achieved before and after reconstructive surgery. Approximately 30% of HD patients will experience at least one episode of HAEC. Note, however, that there is currently no consent on the exact definition of HAEC [1,4].

According to the ERNICA guideline, patients should receive rectal irrigations 1–3 times a day to decompress the bowel until the definitive pull-through operation is performed, which can be achieved in 75% of patients [1]. A survey of European clinicians found that 30% would create an ostomy awaiting pull-through surgery [5].

The purpose of this study is to describe the standard protocol of a specialized institution and present a descriptive study of patients over two years to review the pre-, peri- and postoperative strategies in patients with standard HD. We describe patient characteristics at presentation, anatomical features, preparation for surgery and our institutional pathway for early perioperative recovery and postoperative rehabilitation.

## 2. Materials and Methods

We conducted a cross-sectional chart review of all patients with HD—excluding total colonic aganglionosis and redo pull-through—who underwent reconstruction between 22 January and 23 December at our center. Ethical approval was obtained from the University of Rostock (A2022-0187 and A2024-0018).

Standard preparation consisted of ensuring adequate histopathological diagnosis, determining the length of the aganglionic bowel segment using a colonic contrast enema, and assessing the patient’s nutritional status. In preparation for pull-through surgery, we used the Rostock Irrigation Protocol described below. Rectal irrigation facilitated adequate decompression, intending to avoid an ostomy.

If a stoma was present, obtaining catheter tolerance and transferring fresh stool into the distal limp prepared the distal bowel and perineum for pull-through.

We advised parents to use a soft silicone Foley catheter to accustom the child to rectal tube insertion. Our preoperative bowel management protocol is shown in Table 1.

### 2.1. Immediate Preoperative Bowel Management

We aim for an effective preoperative washout before the pull-through surgeries. Subsequently, the washout the day before surgery was conducted by our medical staff to secure adequate decompression.

### 2.2. Pull-Through Surgery Operative Approach—Swenson Type

The operative technique used for all children is a laparoscopic-controlled transanal pull-through, leaving 1–2 cm of muscle.

### 2.3. Postoperative Bowel Management

Enhanced recovery after surgery (ERAS) is applied to all patients. This includes rapid feeding, good pain relief, and early mobilization. Routine transanal irrigation is used in the event of abdominal distension and is continued for the rehabilitation period.

We actively avoid routine anal dilatations. Three months after the pull-through surgery, we perform one digital examination to palpate the anastomosis in the outpatient clinic. The institutional protocol is shown in Table 2.

The definition used in this study for **Hirschsprung-associated enterocolitis (HAEC)** is the Frykman modification of the Pastor score, with a cut-off value of 4 [1,4]. The HAEC score contains information on patient history, physical and radiological examination, and laboratory results, with a maximum value of 20 [1,4].

### 2.4. Perianal Skin Condition

**Figure 1:**
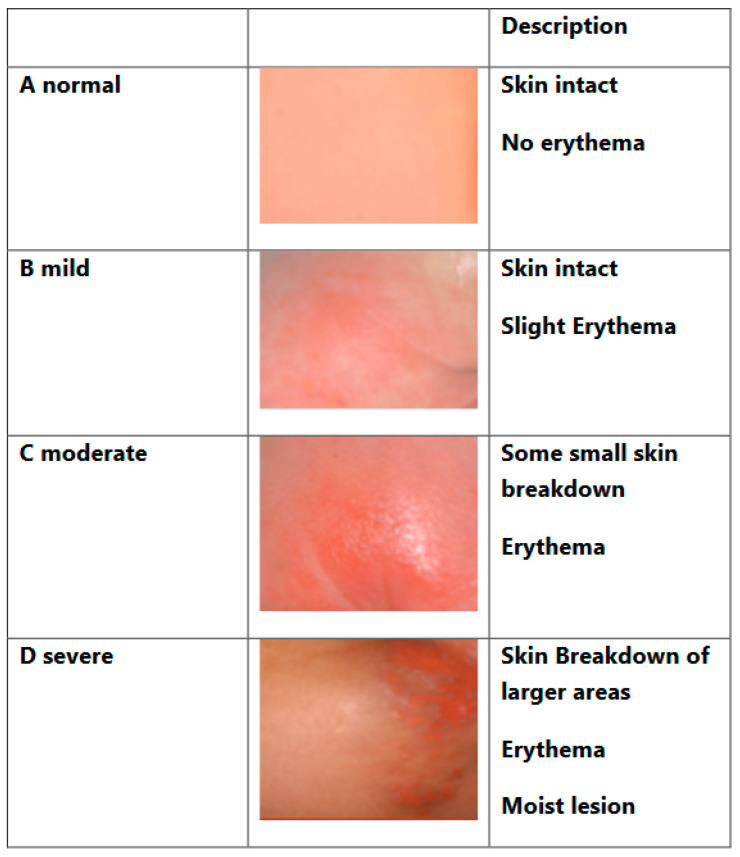
Perineal skin condition. Adapted from the Nappy Rash Protocol, Great Ormond Street Hospital, London.

We used the definition visualized in Figure 1 to provide an objective way of assessing the perianal condition using a standardized grading system. Standardization facilitates communication between nurses and doctors and between families and the medical team.

### 2.5. Rostock Irrigation Protocol Used for Washouts

*Material*: We use a very soft silicone Foley catheter (12–16 French depending on age and size), a 60 cc syringe and two bowls. Vaseline is used to lubricate the catheter.

*Washout fluid: hand-warm* tap water is safe for all ages in a country like Germany, with well-controlled tap water also suggested by clinicians in Europe and the USA [6].

*Volume*: We do not use calculations to assess the correct amount of water and there is no upper limit as described by de la Torre [7]. The endpoint of successful irrigation is reached when the water flowing back is transparent and the child’s abdomen is very flat, almost below the level of the thorax.

*Frequency*: usually 1–2 irrigations per day will allow for sufficient decompression and an additional irrigation should be performed if necessary.

*Technique*: The Foley catheter is initially inserted 5–10 cm to allow for the decompression of gas and stool. To facilitate catheter advancement, it is sometimes helpful to introduce water during insertion. The syringe is disconnected, and stool and gas will passively flow back. A small amount of water (20 mL aliquots) is injected and the catheter is then disconnected again to allow the stool and gas to flow back. Sometimes, stool can be aspirated. Next, the catheter should be moved forward, i.e., more proximally. This helps to evacuate trapped air and feces. Subsequent bowel massage helps to ensure effective decompression and reassures the child. This is repeated until the backflow is clear and the waste pot is filled with the amount of water installed.

*Position*: Newborn babies usually tolerate rectal irrigation in the supine position. Older children may prefer to sit, in which case we insert the catheter, close the nappy, and irrigate in a sitting position.

### 2.6. Bowel Management Protocol after Pull-Through for Hirschsprung Disease

Table 2 shows the bowel management protocol after successful surgery.

**Table 2:** Bowel management protocol at Colorectal Center Rostock—after pull-through for Hirschsprung disease.

	All	Too Much Stool	Perianal Skin Excoriation	Enterocolitis	Constipation
Perianal Skin Protection		-Barrier cream -Increase TAI	-Ilex^®^ barrier cream -Increase TAI		
Perianal Hygiene	Showering after defecation, using water to rinse, e.g., HappyPo ©,and no wiping				
Dietary Advice	Low-fiber diet	Stool thickener, early carrot, and psyllium husks	Early carrot		Low-fiber diet
Transanal Irrigation (TAI)	If no spontaneous defecation, ideally advised in postoperative period	Increase irrigation to 2–3 times daily	Increase irrigation to 2–3 times daily	Increase irrigation to 2–3 times daily	If no spontaneous defecation, at least once daily
Microbiome	Probiotics				
Antibiotics	Only in case of enterocolitis			Metronidazole oral	
Laxatives					Macrogol-based laxatives

### 2.7. Data Management and Statistics

The data were retrieved from electronic medical records, clinical reviews and discharge letters and entered into an Excel sheet. After data cleaning, the data were transferred to SPSS, and an analysis was performed with SPSS 26.0. We analyzed the data for the total group and the two subgroups (A no stoma; B stoma). The results were expressed as the mean and standard deviation or the median and range. The analysis of the groups was performed using t-test or chi-square depending on the variables. A linear-by-linear association model was applied for nominal contingency chi-square tests. The Fischer exact test was used for categorical variables. A *p*-value of <0.05 was considered significant.

## 3. Results

A total of 132 children with HD were seen in our colorectal clinic during the study period. In total, 47 children with any form of HD underwent pull-through surgery during the 2-year study period. We excluded 15 children with total colonic HD who underwent pull-through surgery during the study period. We excluded one child with redo pull-through surgery in standard HD. Subsequently, only children with standard HD and primary pull-through (*n* = 31) were included in our institutional pathway analysis. We describe the pre-, intra- and postoperative data.

Our group consisted of 26 boys and 5 girls. The median age at pull-through surgery was 9 months. In total, 19 (59.3%) had already had a sufficient external rectal biopsy at the time of presentation to our center. A total of 12 (38.7%) children presented with an insufficient biopsy or no biopsy yet, so we performed a rectal biopsy. All children had a diagnostic rectal biopsy confirming aganglionosis. All children had a contrast enema to determine the length of the aganglionic segment.

Table 3 shows the characteristics of the children with HD for the whole cohort and distinguished between children with no stoma and with an existing stoma. There was no significant difference in the affected length of aganglionosis. However, we note a significant difference in the anatomical level of HD. In total, 23/31 (75%) had no stoma at the time of consultation and all were using rectal washouts to decompress the bowel. A total of 8/31 (25%) children had a stoma, of whom 4 (50%) had a colostomy and 4 (50%) had an ileostomy.

Table 4 shows the perioperative patient data for all children undergoing pull-through during the study period. We noted 6.5% involved complications, with one unplanned stoma in each of the groups. One patient with no prior stoma developed a fever after discharge and was taken to another hospital where a stoma was performed due to a potential wound-healing problem at the rectal anastomosis. Another patient with a prior stoma and pull-through with reversal of the colostomy deteriorated 5 days after surgery, with signs of systemic infection. On explorative laparotomy, the colostomy reversal was unremarkable and there was a potential wound-healing disturbance at the rectal anastomosis dorsally.

Early postoperative bowel management was followed by a structured postoperative follow-up and the monitoring of bowel function, perineal skin condition, overall growth development and episodes of enterocolitis. We saw our children 7–10 days, 6 weeks and 3 months after discharge and then every 6 months thereafter, usually extending to an annual follow-up once toilet training was achieved. Follow-up was carried out both in person and by means of telemedicine. This is especially useful if the child is doing well and the family lives far away. In addition, we provided a low-threshold, easy way for the families to communicate in case they have any questions.

**Figure 2:**
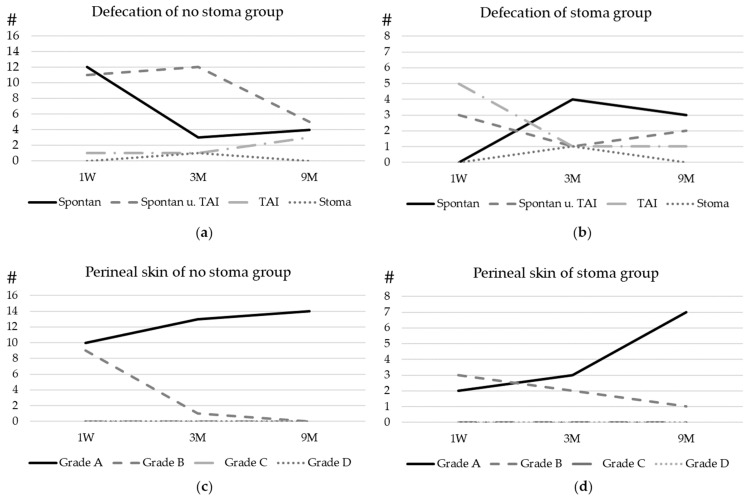
Postoperative bowel function and bowel management: the development of defecation of patients with (**a**) no stoma before surgery and (**b**) a stoma before surgery. (**c**) The development of the perineal skin condition in patients with no prior stoma and (**d**) the development of the perineal skin condition in patients with a prior stoma. For the graduation of the skin, please refer to Figure 1. Spontan = spontaneous defecation, TAI = transanal irrigation, and Stoma = defecation through stoma. Y-axe # Number of patients.

The follow-up is visualized in Figure 2. We did not have a significantly (*p* = 0.04) higher rate of nappy rash 3 months after pull-through in children who had a stoma before pull-through.

## 4. Discussion

Preoperative preparation paves the way for intraoperative surgical reconstruction in primary pull-through surgery for HD. The ability to achieve effective decompression through the use of washouts can help to avoid the need for a stoma. The pediatric surgical community has moved to avoid stomas and aims to create washouts whenever possible in preparation for pull-through surgery [8]. Avoiding a stoma facilitates a technically easier pull-through for the surgeon, with a shorter operative time and faster recovery. Furthermore, stoma-related problems described in up to 30% of children with a stoma with a background of Hirschsprung [9] are avoided. More importantly, recovery in terms of regaining enteral intake and bowel function is significantly faster, as demonstrated by comparing the two groups with and without a stoma in our cohort treated in the same colorectal unit by the same team.

An intact dentate line, anal canal and anal sphincter are essential for continence. Structured postoperative bowel management supports postoperative recovery and the attainment of social continence [10].

### 4.1. Child without a Stoma—Preoperative Bowel Management to Prepare for Surgery: PREHABILITATION

Ideally, daily washouts by the family will allow for adequate decompression and prevent enterocolitis. In addition, effective washouts will relieve the obstruction and keep the colon decompressed. An adequate washout helps reduce an initially dilated prestenotic bowel segment and facilitates smooth anastomosis [7].

We emphasize the importance of educating the family to perform an adequate and effective washout. Not only should we aim to avoid an unnecessary procedure, but we also aim to see the smoother recovery of children who do not have a stoma.

### 4.2. Child with a Stoma: Preoperative Situation

Stool is evacuated through the stoma until pull-through surgery, leaving the aganglionic and adjacent bowel uninvolved in the circulation, i.e., subsequently unused mucosa.

Children with a preoperative stoma are more likely to develop a perianal rash/skin excoriation despite thorough preparation with stool transfer to the distal limb. In total, 40% of children with a stoma report having a perianal rash 3 months after pull-through surgery, compared with only 7% of children who did not have a stoma before pull-through surgery. Perianal excoriation can be managed through adequate perineal care, bowel management and the use of various barrier creams [1].

Even with this thorough preparation, children with a stoma pass stool later than their counterparts without a stoma and are significantly slower to achieve full enteral nutrition (*p* < 0.001).

### 4.3. Perioperative Bowel Management

Rapid recovery using an ERAS protocol has significantly changed the perioperative and early postoperative management of children undergoing abdominal surgery [11,12]. In particular, the transanal approach avoids intra-abdominal dissection and early feeding is recommended [13]. In our cohort, all children had their first enteral feed on the day of surgery regardless of a previous stoma; however, we noted a significantly faster full enteral intake in children without a previous stoma (*p* < 0.001).

Bearing in mind that pain and stress can affect bowel motility, which is also disturbed by the technical act of surgery, both can lead to temporary postoperative bowel paralysis.

In total, 78% (N = 25) of children in our cohort passed stool within the first 24 h and the remaining within the second day.

Immediately after surgery, some children have difficulty passing air, and rectal irrigation can relieve symptoms of bloating and abdominal distension.

Similarly, Zhang et al. show that rectal irrigation not only reduces abdominal distension but also prevents postoperative HAEC [14]; we did not see any HAEC in our direct postoperative observation. Controversially, Beltmann et al. describe a likely mortality due to perforation when early postoperative irrigation is used; in a cohort of 106 pull-through operations within a 15-year period in which 22% had postoperative complications anyway [15].

Unpublished data from our department show that children tolerate rectal irrigation well if introduced smoothly in a child-friendly, relaxed environment before surgery. In order to support the adaptation and recovery of the pull-through, we use early transanal irrigation as physiotherapy for the bowel.

### 4.4. Postoperative Bowel Management: REHABILITATION

(a) Developing spontaneous bowel movement

We see an overall good potential for spontaneous bowel movement in the very early recovery period, with 52% of all children passing stool spontaneously, but after 3 months, 90% need at least supportive rectal irrigation to pass stool effectively and can be weaned off irrigation over time. By 9 months, 44% are passing stool spontaneously and this figure is now increasing steadily. At 2-year follow-up, the majority defecates spontaneously.

This highlights the importance of incorporating postoperative transanal irrigation into routine bowel physiotherapy. Systematic irrigation not only reduces the risk of enterocolitis but also helps children to eventually become socially clean.

In older children, we progress to active transanal irrigation as needed. We use hydrosonographic ultrasound to estimate the amount of water needed. We monitor the success of the bowel management program by assessing fecal load through ultrasound [16]; both sonographic techniques have the advantage of sparing ionizing radiation, which is often described in other bowel management programs [17].

(b) Overcoming perianal rash

It is not uncommon to have frequent bowel movements with small portions during the early recovery period. These low-volume bowel movements are partly caused by the removal of the obstructive pressure and partly by the perioperative antibiotics, which affect the consistency of the stool. Too many stools, especially small ones, cause perianal rash. In children with a stoma, 3 months after pull-through, we note significantly (*p* = 0.04) more cases of nappy rash. In these cases, bowel management with transanal irrigation can reduce the frequency of defecation and thus improve the nappy rash.

(c) Overcoming outlet obstruction

Rehabilitation is a well-accepted concept in musculoskeletal surgery or surgery for other functional impairments. The pull-through bowel not only needs to find its new position but also needs to learn to function as a neorectum. The healthy ganglionic bowel representing the prestenotic dilatation will become the neorectum once anastomosed to the remaining small native rectum, overcoming a size mismatch. The pull-through bowel will take over its function and will regain an adequate peristalsis, relieved from the functional obstruction caused by the aganglionic segment.

Outlet obstruction characterized by the persistent absence of the rectoanal inhibitory reflex (RAIR) can be a functional symptom after an otherwise successful pull-through. However, mechanical complications such as twist, stricture or persistent aganglionosis should be ruled out and, if present, appropriately treated [17,18,19,20].

We observe, as do others, that maturing children learn to overcome the obstruction of a non-relaxing internal sphincter and grow out of the outlet obstruction that is presented as smearing and sometimes mistaken for fecal incontinence [13,21,22]. However, Fosby et al. reports no improvement with age in a Norwegian cohort [23].

Other groups have successfully used Botox to relieve this well-known temporary outlet obstruction, which can be repeated if necessary [24]. However, in our experience, using transanal irrigation as active physiotherapy trains the distal bowel.

### 4.5. Education and Support for Families

Families can be overwhelmed by bowel management, especially when faced with a new diagnosis of Hirschsprung [2,3]. Educating parents and involving them as early as possible in the management of rectal irrigation alongside the medical team builds parental confidence [1,10]. Families benefit from being able to raise their questions and concerns at any time with a low threshold. E-mail, telephone and video calls can be used as a supplement, especially for families living further away [2].

Looking at the experience of affected families, a Dutch nationwide study reveals that if the family is familiar with Hirschsprung, not only does the likelihood of ongoing bowel management increase but also the families are more likely to use transanal irrigation as their preferred method of bowel management in both rectosigmoid and long-segment Hirschsprung. This familiar coping positively influences the patients coping [25].

### 4.6. Strength and Limitations

We describe our standardized bowel management protocol by analyzing a cohort treated by the same team within a short time period. This means that there are no confounders by the overall medical advancements. Monitoring data in a clinical environment is challenging, even if the patients are followed prospectively, as sometimes not all information is sufficiently recorded.

## 5. Conclusions

The success of pull-through surgery is supported by preoperative bowel management and structured follow-up, with bowel management as physiotherapy.

Avoiding a stoma in the first place not only avoids surgery but also allows for a faster recovery after pull-through surgery. If a stoma is present, careful preparation will help to prepare the distal bowel, although postoperative recovery of bowel function will take longer than in patients without a stoma, despite the best preparation.

Rectal irrigation is helpful in the postoperative period to facilitate a smooth recovery and to support postoperative recovery, serving as physiotherapy for the bowel.

## Figures and Tables

**Table 1:** Bowel management protocol at the Colorectal Center Rostock—preparation for pull-through surgery in Hirschsprung disease.

	No Stoma	Stoma Present
**Perianal Skin Protection**	Regularly rectal irrigation; bring the perineum in contact with stool.	Transfer fresh stoma effluent to distal limp (at least for a minimum of 4 weeks prior to pull-through).
**Perianal Hygiene**	Shower after defecation; use water to rinse, e.g., HappyPo ©. No wiping.	
**Dietary Advice**	Ideally keep on breastmilk till pull-through.	Ideally keep on breastmilk till pull-through. If older, follow a low-fiber diet.
**Acquisition of Tolerance to Catheter**	Acquired with ongoing rectal irrigation.	Insert rectal tube intermittently.
**Microbiome**	Probiotics only if not breastfed.	(a) Probiotics if not breastfeeding; (b) Transfer fresh stoma effluent to distal limp.
**Antibiotics**	Not routinely.	Not routinely.

**Table 3:** Characteristics at presentation.

	All *n* = 31	Group A No Stoma *n* = 23	Group B Prior Stoma *n* = 8	*p*-Value
Sex (*n*)	Male: 26	20	7	ns
	Female: 5	4	1	
Additional anomalies (*n*)	No: 26	21	5	ns
	Yes: 5	2	3	
	Trisomie 21: *n* = 3	1	2	ns
	If other, which: Cardiac: 2 Duodenal Atresia: 1	2	1	
Age at contrast (months)	Mean: 8.3	8.8	6.8	ns
	Median: 6.5	6.5	6	
	Range: 1–26	1–26	1–13	
Transition zone on contrast (*n*)				*p* = 0.039
Type 1	3	3	0	
Type 2	22	18	4	
Type 3	5	2	3	
Type 4	0	0	0	
Aganglionic lengths on contrast (cm)	Mean: 8.7	8.1 ± 2.5	12.3 ± 2.5	*p* = 0.07
	Range: 5–15	5–12	10–15	
Type of stoma			Ileostomy: 4/8 Colostomy: 4/8	
Age at rectal biopsy (months)	Mean: 8.2	8.8	2.5	ns
	Range: 1–26	1–26	1–4	

Transition zone on contrast: Type 1 = rectum, Type 2 = rectosigmoid, Type 3 = colon descendens to left flexure, and Type 4 = up to colon ascendens. ns—non significant.

**Table 4:** Perioperative bowel management.

	All *n* = 31	Group A No Prior Stoma *n* = 23	Group B Prior Stoma *n* = 8	*p*-Value
Age at pull-through (months) (*n* = 36)	Median: 9.5	9	9	ns
	Mean: 15.4	15.9 ± 19.8	11.8 ± 7.8	ns
	Range: 4–97	4–97	5–27	ns
Weight at pull-through (kg and percentile) (*n* = 36)	Mean: 9.4, P33 Range: 7–12, P0.5–94	9.6, P32 7–12	8.9, P31 7.2–11.6	ns
Operative time of pull-through (h) primary pull-through	Mean: 2:41	2:24	2:50	ns
	Range: 1:26–4:27	1:26–4:27	2:37–3:57	
Primary pull-through (*n*)	31	23	8	
Rectal irrigation prior to pull-through (*n*)	23	23	0	
Stool transfer to distal limp (*n*)	8	Not applicable	8	
Training catheter tolerance (*n*)	8	Not applicable	8	
Length of resected segment during surgery (cm)	Mean: 13.8	12.8	15.8	ns
	Range: 5–28	5–25	7–28	
Protective stoma after pull-through (*n*)	0	0	0	
Unplanned stoma within 30 days (*n*)	2	1	1	ns
Clear fluids on operative day (*n*)	31	23	8	
Slow establishment of enteral intake; no full enteral feeds after 48 h (*n*)	5	1	4	*p* < 0.001
Time to first spontaneous defecation (*n*)	24 within 24 h	22	3	
	7 within 24–48 h	1	5	
Time to rectal irrigation (*n*)	Performed on day 1 post-op		5	
30-day complication occurrence (*n*)	2	1	1	ns
Rectal dilatation (*n*)	0	0	0	

ns—non significant.

## Data Availability

The data presented in this study are available on request from the corresponding author. The data is recorded in our Rostock Colorectal Hirschsprung patient registry due to privacy or ethical restrictions.

## Abbreviations

ERAS	Enhanced recovery after surgery
HAEC	Hirschsprung-associated enterocolitis
HD	Hirschsprung disease
ns	Not significant
RAIR	Rectoanal inhibitory reflex
TAI	Transanal irrigation
